# Essential Oils and Hydrolates: Potential Tools for Defense against Bacterial Plant Pathogens

**DOI:** 10.3390/microorganisms10040702

**Published:** 2022-03-24

**Authors:** Maria Rita Proto, Enrico Biondi, David Baldo, Matilde Levoni, Gianfranco Filippini, Monica Modesto, Maura Di Vito, Francesca Bugli, Claudio Ratti, Paola Minardi, Paola Mattarelli

**Affiliations:** 1 Dipartimento di Scienze e Tecnologie Agro-Alimentari, Università di Bologna, Viale G. Fanin 44, 40127 Bologna, Italy; david.baldo@unibo.it (D.B.); matilde.levoni@studio.unibo.it (M.L.); gianfranco.filippini@unibo.it (G.F.); monica.modesto@unibo.it (M.M.); claudio.ratti@unibo.it (C.R.); paola.minardi@unibo.it (P.M.); paola.mattarelli@unibo.it (P.M.); 2 Dipartimento di Scienze Biotecnologiche di Base, Cliniche, Intensivologiche e Perioperatorie, Università Cattolica del Sacro Cuore, Largo A. Gemelli 8, 00168 Rome, Italy; wdivit@gmail.com (M.D.V.); francesca.bugli@unicatt.it (F.B.); 3 Dipartimento di Scienze di Laboratorio e Infettivologiche, Fondazione Policlinico Universitario A. Gemelli IRCCS, Largo A. Gemelli 8, 00168 Rome, Italy; 4 Bologna University Scardovi Collection of Bifidobacteria BUSCOB, Dipartimento di Scienze e Tecnologie Agro-Alimentari, Università di Bologna, Viale G. Fanin 44, 40127 Bologna, Italy

**Keywords:** essential oil, hydrolate, antibacterial activity, induced resistance, sustainable agriculture defence

## Abstract

The essential oils (EOs) of *Origanum compactum* and *Satureja montana* chemotyped (CT) at carvacrol, two *Thymus vulgaris* CT at thujanol and thymol, and Hydrolates (Hys) of *S. montana* and *Citrus aurantium* var. *amara* were chosen for studying their bactericidal efficacy against few phytobacterial pathogens. The Minimal Inhibitory Concentration (MIC) and Bactericidal Concentration (MBC) were found by microdilution assay. The essential oils of *O. compactum* (MBC 0.06% *v*/*v*), *T. vulgaris* CT thymol (MBC 0.06% *v*/*v*), and Hy of *C. aurantium* (MBC 6.25% *v*/*v*) resulted in being the most effective against *Erwinia amylovora*; thus, they were used as starting concentrations for ex vivo assays. Despite the great in vitro effectiveness, the disease incidence and the population dynamic ex vivo assays showed no significant results. On the other hand, EO of *O. compactum* and Hy of *C. aurantium* (at 0.03% and 4.5% *v*/*v*, respectively) showed resistance induction in tomato plants against *Xanthomonas vesicatoria* infections; both treatments resulted in approximately 50% protection. In conclusion, EOs and Hys could be promising tools for agricultural defense, but further studies will be necessary to stabilize the EOs emulsions, while Hys application could be an effective method to prevent bacterial diseases when used as resistance inducer by pre-transplantation treatment at roots.

## 1. Introduction

Essential oils (EOs) are mixtures of complex volatile compounds, which are synthesized through secondary metabolic pathways in several plant species, particularly aromatic plants [1]. EOs are normally obtained by hydro-distillation or steam distillation of plant tissues and by cold pressing *Citrus* spp. fruit peels; the co-product of the distillation is the hydrolate (Hy), which represents aromatic water containing approx. 0.1% EO compounds [2,3].

In nature, EOs play important roles in plant defense, acting as antimicrobials and as signal molecules to communicate with other plants and beneficial insects [4,5,6].

The biological properties of medicinal plants are known since ancient times thanks to folk medicine; in the last years, these properties have been rediscovered and the mechanisms of action have begun to be studied, especially concerning their antimicrobial properties, as well as healing properties (i.e., analgesic, sedative, anti-inflammatory, spasmolytic); their use in the cosmetic and food industry as preservatives is increasing [7,8,9]; in addition, the recent findings on the effectiveness of EOs against antibiotic-resistant bacteria, are very relevant [3,10,11].

In agriculture, the defense against plant bacterial pathogens is provided by preventive plant treatments with chemicals mostly based on copper compounds. The use of copper compounds is advantageous for their high toxicity towards plant pathogens, low toxicity in mammals, low costs, and prolonged effect due to their stability. On the other hand, their excessive use, since they were discovered, has caused several negative effects, the most relevant of which are represented by the accumulation in the soil and the selection of copper-resistant strains. From 2019, the European Union imposed strong restrictions on the use of copper-based compounds [12], limiting its use up to 28 kg/ha over 7 years. In organic farming, the use of these compounds is still allowed, although some European countries, such as Denmark and Switzerland, have already banned their use as protection products [13]. Moreover, in all continents but Europe, bacterial plant diseases are also controlled through the application of antibiotics (e.g., streptomycin, gentamycin), which brings negative effects such as bacterial resistance, fruit residues, and accumulation in the soil [14,15].

In this scenario, it is necessary to find alternatives to copper or to optimize its use; thus, the application of EOs and/or Hys could be an effective and environmentally sustainable defense method for plant health and, consequently, for human health.

In the last decade, the literature on the use of EOs for the defense against phytopathogens has increased, especially on in vitro activity [16,17,18,19], although there is still not enough uniformity on the protocols used, consequently it is difficult to compare the results of different studies. However, there are still few studies *in planta* and ex vivo. The present study aimed to investigate the efficacy of several EOs and Hys (i) through in vitro tests against different bacterial plant pathogens such as *Pseudomonas savastanoi pv*. *savastanoi* (the causal agent of the bacterial canker of olive), *Allorhizobium vitis* (agent of the grape crown gall), *Erwinia amylovora*, and *Xanthomonas vesicatoria*; (ii) through ex vivo tests against *E. amylovora* (etiological agent of fire blight of pomaceous plants) to evaluate their efficacy in preventing disease symptoms on pear flowers and fruitlets when directly applied against the pathogen and in reducing *E. amylovora* epiphytic population on apple flowers; (iii) through in planta tests on tomato plants, to evaluate the EOs’ and Hys’ ability to induce plant defense responses against *X. vesicatoria* (causal agent of Bacterial Leaf Spot of Tomato, BLST).

## 2. Materials and Methods

### 2.1. Essential Oils and Hydrolates

The EOs used in this study were: *Origanum compactum* chemotyped (CT) carvacrol (OC), *Thymus vulgaris* CT thymol (TM), and CT thujanol (TJ) provided from Pranarôm (Colognola ai Colli (VR), Italy); *Satureja montana* CT carvacrol (STG) and *S. montana* hydrolate (HySTG) obtained from Gadoi (Badia Calavena, Verona, Italy); and the hydrolate of *Citrus aurantium* var. *amara* (AA) obtained from Magentina (Poirino (TO), Italy). All EOs were used as emulsions while Hys were used as they were. The chemical analysis of EOs and Hy of *S. montana* were provided by the producers (Tables S1–S5). The chemical composition of Hy of *Citrus aurantium* is described in Di Vito et al. (2018) [20].

### 2.2. Essential Oils Emulsions

The EOs emulsions were prepared using Tween 80 according to the following two methods.

Method 1 (M1): the stock emulsion was prepared with 1% Tween 80 and 1% EO; both were placed in an Eppendorf tube and vortexed for approx. 1 min; then, sterile distilled water (SDW) or growth medium (LB: 10 g tryptone, 5 g yeast extract, 10 g NaCl, in 1 L distilled water, pH 7.0) was added and vortexed again for at least 3 min, after which a milky to opaque emulsion was obtained.

Method 2 (M2): The stock emulsion was prepared by placing 1% Tween 80 and 1% EO in a 50 mL Schott bottle put onto a heating magnetic stirrer (≤30 °C) at maximum speed, adding SDW until a clear or slightly opaque solution was obtained. The M2 procedure was used only for ex vivo and in planta experiments on pear fruits and tomato plants, respectively.

### 2.3. Bacterial Cultures

The wild-type PHS-ER 1077.7/94 (ex OMP-BO 1077.7/94) and rifampicin-resistant *E. amylovora* 273R1 strains of *E. amylovora* were routinely grown at 27 °C for 24–48 h on King’s B medium (KB) [21] and KB amended with 20 ppm rifampicin, respectively. *X. vesicatoria* strain DISTAL 2684, *A. vitis* strain DISTAL 5159, and *P. savastanoi* pv. *savastanoi* strain DISTAL 11,628 were routinely grown at 27 °C for 48–72 h on Glucose, Yeast extract, CaCO_3_ (GYCA) [22], Yeast Mannitol Agar (YMA) [23], and KB, respectively. For in vitro and ex vivo assays, 0.1 OD_600nm_ (approx. 10^8^ CFU/mL) bacterial cells aqueous suspensions obtained by 24 h old axenic bacterial cultures were performed; the suspension was then diluted 1/100 (approx. 10^6^ CFU/mL). A vitality assay to confirm the bacterial concentration of inoculum was performed.

For *in planta* trials on tomato plants, 0.1 OD_600nm_ (approx. 10^8^ CFU/mL) *X. vesicatoria* DISTAL 2684 suspension in SDW obtained by 24 h-old axenic bacterial culture was performed; the suspension was then diluted 1/10 (approx. 10^7^ CFU/mL). A vitality assay to confirm the bacterial concentration of inoculum was performed.

### 2.4. In Vitro Experiments

#### 2.4.1. Microdilution Assay

The minimum inhibitory concentration (MIC) and the minimum bactericidal concentration (MBC) of EOs and Hys were evaluated with microbroth dilution assay according to EUCAST International Guidelines [24], slightly modified; a bacterial inoculum of approx. 2 × 10^6^ CFU/mL and Luria Bertani medium (LB) were used. The MIC test was performed on a 96-well microtiter plate: aliquots of 50 μL of EO emulsion, prepared in LB medium, or Hy were added to 50 μL of bacterial suspension made in the same medium. M1 has been used for the preparation of the EO emulsion. EO’s effectiveness was tested using two-fold scalar dilutions between 1% (10 mL/L) to 0.002% *v*/*v* (0.02 mL/L). The concentrations of the hydrolates ranged from 50% (500 mL/L) to 0.1% *v*/*v* (1 mL/L).

LB broth, added with and without 1% Tween 80, were used as positive controls and LB with 100 µg/mL streptomycin sulfate (SM) as antibacterial activity control. The microplates were sealed with an adhesive film (to avoid dispersion of the EO more volatile components) and incubated for 24 h at 27 °C. After incubation, the bacterial growth was evaluated spectrophotometrically at 620 nm (Multiskan EX, Thermo Fisher Scientific Oy, Vaanta, Finland). To assess MBC values, 10 μL were taken from the well showing no microbial growth and seeded onto specific growth medium agars; after the incubation time required for each strain, CFU/mL were counted to assess cell viability.

The MIC is defined as the lowest concentration that inhibits 99% of the bacterial growth; the MBC is defined as the lowest concentration resulting in the death of 99.9% or more of the initial inoculum [25]. Each assay was performed in triplicate.

#### 2.4.2. Macrodilution Assay

The bacterial aqueous suspension (150 μL, approx. 10^8^ CFU/mL) was added to 15 mL of LB broth, in 50 mL Falcon tubes, with MIC and MBC concentrations of EOs obtained in microbroth dilution assay. The suspension was inoculated in LB broth alone or added with SM at 100 μg/mL as negative and positive control, respectively. The tubes were placed in a rotary incubator at 80 rpm, at 25 °C for 24 h; CFU/mL were counted by serial dilutions to assess cell viability. Each assay was carried out in triplicate.

### 2.5. Disease Incidence Experiments

#### 2.5.1. Fire Blight Control on Pear Flowers

Freshly opened pear flowers cv. Williams (6 flowers × 4 replicates/treatment) were kept, after detachment, in 1.5 mL Eppendorf tubes containing 1 mL sucrose solution (10%) [26]. The EO treatments were prepared according to M2; 30 µL of OC 0.06%, OC 0.12%, and TM 0.06% were dropped directly onto the hypanthium of each flower; then the flowers were sealed into Plexiglas boxes (100% relative humidity, RH; humid chamber), and maintained in a climatic chamber at 25 °C (16 h light, 8 h darkness). After 24 h, the treated flowers were inoculated with an aqueous suspension of PHS-ER 1077.7/94 *E. amylovora* strain (30 µL; approx. 10^6^ CFU/mL) onto the hypanthium, as well and incubated as previously described. Phytopathometric assessments were daily carried out up to 6 days post-inoculation (dpi). Disease incidence (DI; Number diseased flowers/Number inoculated flowers%) was evaluated, and data were elaborated with ANOVA tests (*p* ≤ 0.05) using SPSS v 15.0 for Windows. The pathogen was directly re-isolated from the ooze of selected diseased flowers on NSA, and it was identified through colony morphology recognition and molecular assay [27].

The SM (100 µg/mL) and SDW were used as positive and negative control, respectively.

#### 2.5.2. Fire Blight Control on Pear Fruitlets

Two experiments were carried out on immature pear fruitlets cv. Abate Fétel (4 pears X 4 replicates/treatments). The first experiment was conducted as previously described by Minardi et al. [28], but with essential oil pre-treatments. Under sterile conditions, a well was dug on the fruitlet surfaces, previously sterilized with 70% ethanol; then, 60 μL of each treatment (Table 1) was deposited into the well. After 1 h (to allow treatment adsorption), 40 μL of aqueous suspension containing the *E. amylovora* strain PHS-ER 1077.7/94 (approx. 10^6^ CFU/mL) was placed into the same well. The inoculated pears were sealed into a humid chamber and incubated in the climatic chamber at 25 °C (16 h light, 8 h darkness). Phytopathometric assessments were carried out daily for up to 6 days after pathogen inoculation; DI was evaluated (Number diseased fruitlets/Number inoculated fruitlets%), and data were elaborated with ANOVA tests (*p* ≤ 0.05) using SPSS v 15.0 for Windows.

In the second experiment, the pear fruitlets were pierced with a sterile needle (3 punctures per fruitlet of about 0.5 cm depth) and sprayed with OC 0.12% (M2). After drying (approx. 1 h), the pear fruitlets were inoculated by spraying the *E. amylovora* aqueous suspension (approx. 10^6^ CFU/mL). Incubation, phytopathometric assessments, and DI were performed as described above. The pathogen was directly re-isolated from ooze of selected diseased fruitlets on NSA, and it was identified through colony morphology recognition and molecular assay [27].

In both experiments, SM (100 µg/mL) and SDW were used as positive and negative control, respectively.

### 2.6. Population Dynamics of E. amylovora on Treated Apple Flowers

The experiments were carried out on detached apple flowers cv. Rome Beauty (5 flowers × treatment per time point) and kept in Eppendorf tubes containing SDW. Freshly opened flowers were sprayed with OC at 0.12 and 0.50% (prepared according to M2); after 24 h, the rifampicin-resistant strain *E. amylovora* 273R1 (approx. 10^6^ CFU/mL) was sprayed on apple flowers. After incubation at 25 °C in a humid chamber, 5 flowers without petals and pedicel were singularly washed in 1.5 mL Eppendorf tubes containing 1 mL 10 mM MgSO_4_ per time point (1, 24, 48, and 96 h after pathogen inoculation). The pathogen population present on each flower was counted by plating each 10-fold dilution on KB with 20 µg/mL rifampicin; after incubation at 27 °C for 24–48 h, the CFU were counted, and the population of the pathogen was calculated for each flower. SDW and untreated/non-inoculated flowers were used as controls [29].

### 2.7. Scanning Electron Microscopy (SEM) Observations on Apple Flowers

Detached apple flowers, treated with EOs and inoculated with strain *E. amylovora* 273R1 as described in Section 2.7, were used for observations at SEM. At each time point (1, 24, 48, and 96 h after pathogen inoculation), the flowers without petals, pedicels, anthers, and filaments (previously removed by scalpel) were inserted in filter paper envelopes, then were fixed with freshly prepared 2.5% glutaraldehyde in phosphate buffer (PBS 0.1 M; pH 7.2) at 4 °C for 24 h. The samples were then washed twice with PBS at 4 °C. Dehydration was carried out sequentially with ethanol at concentrations of 20%, 30%, and 50% for 7 min each, followed by a 90% ethanol wash for 14 min and then a 100% ethanol wash for 10 min, repeated twice. Samples were subjected to Emitech K850 Critical Point Drying (CPD) using liquid CO_2_ as transitional fluid. The dried samples were placed on 1/2″ slotted head-1/8″ pin stubs covered with conductive 3M 465kp scotch tape and gold-coated using Emitech K500 sputter at 40 mA for 3 min. Observations were made with Philips SEM 515 at 20 kV, 9–13 mm Working Distance, Spot size 20 nm, and SEM micrographs were acquired at 2000 lines 8 ms with a reflex camera Canon 7D in Bulb Mode. Flowers treated with SDW, and untreated and not inoculated flowers were used as controls.

### 2.8. Induced Resistance in Tomato Plants against Bacterial Leafspot

Under climatic chamber conditions, two experiments were carried out on tomato plants cv. VF-10, disposed in randomized blocks (3 plants × 4 blocks/treatment). The tomato plants grown in pots were uprooted at the third and fourth leaf stages, and the root apparatus was soaked for 10 min in 300 mL of OC (at 0.015 and 0.03%) and AA (at 2.2 and 4.5%), prepared according to M2; then, the plants were put back into the pots. After 48 h from the treatment, the plants were experimentally inoculated by spraying the leaf surfaces with an aqueous suspension containing the strain DISTAL 2684 of *X. vesicatoria* (approx. 10^7^ CFU/mL); the tomato plants were then sealed in polyethylene (PE) bags for one day to favor the water congestion and to allow pathogen penetration [30]. The disease assessments were carried out by counting the leaf spots (on 4 leaves/plant) 21 days after pathogen inoculation. Until the disease assessment, the plants were kept in the climatic chamber with a 16 h photoperiod and relative humidity (RH) of 70–75% at 30 °C and 24 °C during day and night, respectively. Data were elaborated with ANOVA test and the relative protection (RP) of each treatment was calculated [(No. leafspots in SDW control plants—No. leafspots in treated plants)/No. leafspots in SDW control plants].

Selected symptomatic leaf samples were used for the isolation and identification of the pathogen. The leaf surface was sterilized with 2% sodium hypochlorite. Necrotic lesions were aseptically collected and crushed into a mortar with 2 mL of sterile distilled water; 30 μL of the 10^−1^ and 10^−2^ diluted extract were dropped onto GYCA medium and incubated up to 48–72 h. Xv-like colonies were sub-cultured and identified with molecular assays [31]. In both experiments, Acibenzolar-S-methyl (ASM; 75 µg/mL, treated 7 d before the experimental inoculation) and SDW were used as positive and negative controls, respectively.

## 3. Results

### 3.1. Essential Oils and Hydrolates

The main components are shown in Table 2 and Table 3; the complete analyses are shown in Supplementary Materials from Tables S1–S5; for *Citrus aurantium* Hy, see Di Vito et al. (2018) [20]. Table 2 shows the main components for each EO. In *T. vulgaris* CT thymol, the reported components are mainly monoterpens with the exception of linalool and β-caryophyllene, which are monoterpenoid alcohol and bicyclic monoterpenoid, respectively. Thymol and carvacrol are phenolic monoterpens, the first one derived from a p-cymene hydride. In the second thyme (CT thujanol), 6 out of 11 major components are alcohols (trans- and cis-thujanol, terpinene-4-ol, linalool, myrcenol, α-terpineol). The reported components are mainly monoterpens except for linalool and myrcenol which are monoterpenoids tertiary alcohols, and for myrcenil acetate which is an ester. Trans- and cis-thujanol are two stereoisomers of monoterpene alcohol. Alpha- and beta- terpinene are two of three isomeric monoterpenes and cyclohexadienes that differ in the positions of their two double bonds. The phenolic monoterpenes carvacrol and thymol, the monoterpene and cyclohexadiene γ-terpinene, and the aromatic monoterpene para-cymene are found in oregano essential oil. The same composition, but in different percentages, is found in the *S. montana* EO, with thymol replaced by α-terpinene.

The main components found in the Hys (Table 3) were, in the case of *C. aurantium*, four monoterpenoid alcohols, linalool, citronellol, geraniol, and nerol (cis-isomer of geraniol); the monoterpenoid ketone carvone; and 2 monoterpenes, terpinolene and α-terpineol (alcohol). In savory, the major components shown in Table 3 are the phenolic monoterpenes thymol and carvacrol.

### 3.2. In Vitro Experiments: Micro and Macrodilution Assays

The EOs of OC, TM, and STG were the most effective against *E. amylovora*, Xv, and *A. vitis*, showing MIC and MBC values between 0.015% and 0.06% *v*/*v*. Against *P. savastanoi*, the lowest MIC and MBCs occurred with OC: 0.03% and 0.015% *v*/*v* for MIC and MBC, respectively. Among the EOs, TJ showed the lowest antibacterial activity towards all pathogens; MBC resulted between 0.125% and 1% *v*/*v*.

The MBC values could be the same or higher in respect to MIC (Table 4). For what concerns Hys, AA showed the higher antibacterial activity towards all strains MBC resulted between 3.1% and 6.25% *v*/*v* with respect to HySTG, whose MBC was 50% *v*/*v*.

The EOs and Hys with the lower MIC and MBC against *E. amylovora* and *X. vesicatoria* were tested in a broth macrodilution to confirm the MBC values.

The MBCs of OC (0.06%), TM (0.06%) and AA hydrolate (6.25%) confirmed their bactericidal activity. The *E. amylovora* population in negative control resulted in approx. 10^9^ CFU/mL. (Supplementary Table S1).

For what concerns the *X. vesicatoria* strain, the MBCs of OC and AA confirmed their inhibitory effect: the *X. vesicatoria* population resulted in being approx. 10^2^ and 10^4^ CFU/mL, respectively, both comparable with the antibacterial activity control (approximately 10^3^ CFU/mL). In the negative control treatment, the bacterial population resulted in 10^8^ CFU/mL (Supplementary Figure S2).

### 3.3. Disease Incidence Experiments: Fire Blight Control on Pear Flowers and Fruitlets

**Figure 1:**
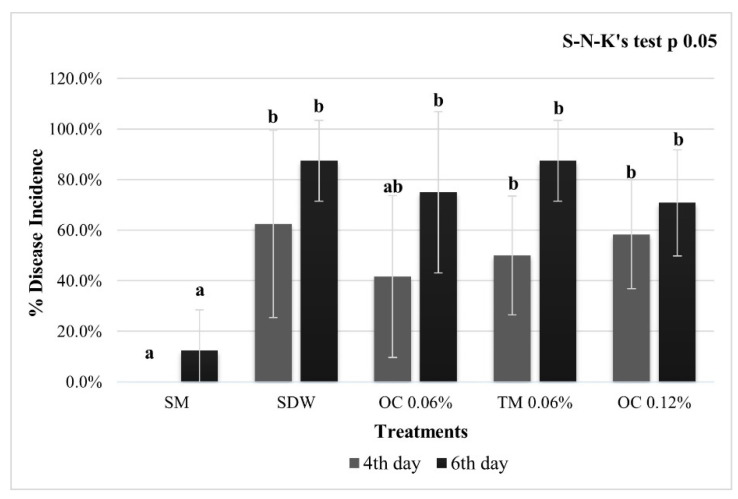
Pear flowers disease incidence. Flowers cv. Williams, inoculated with *E. amylovora*, pre-treated with streptomycin sulphate at 100 μg/mL (SM, positive control), sterile distilled water (SDW, negative control), *O. compactum* CT carvacrol 0.06% and 0.12% (OC 0.06%), *T. vulgaris* CT Thymol 0.06% (TM 0.06%). The phytopathometric assessments were performed 4 and 6 dpi. The bars represent the standard deviations, and the letters indicate the statistical categories (SNK, *p* < 0.05).

On pear flowers, none of the treatments with EO were able to significantly reduce the incidence of fire blight; after 4 days from inoculation, the lowest DI (approx. 42%) was evaluated on flowers treated with OC at 0.06%, while in the OC treatment at 0.12% and TM at 0.06%, the incidence of fire blight was approx. 50% and 58%, respectively, comparable to that evaluated on the control flowers (approx. 62%). After 6 dpi, the treatments of OC 0.06%, TM 0.06%, and OC 0.12% showed a DI of approx. 75%, 87%, and 71%, respectively, similar to the negative control (approx. 87%). On flowers treated with SM, the DI evaluated at 4 and 6 dpi, resulted significantly lower (approx. 0% and 13%) with respect to all treatments (Figure 1).

**Figure 2:**
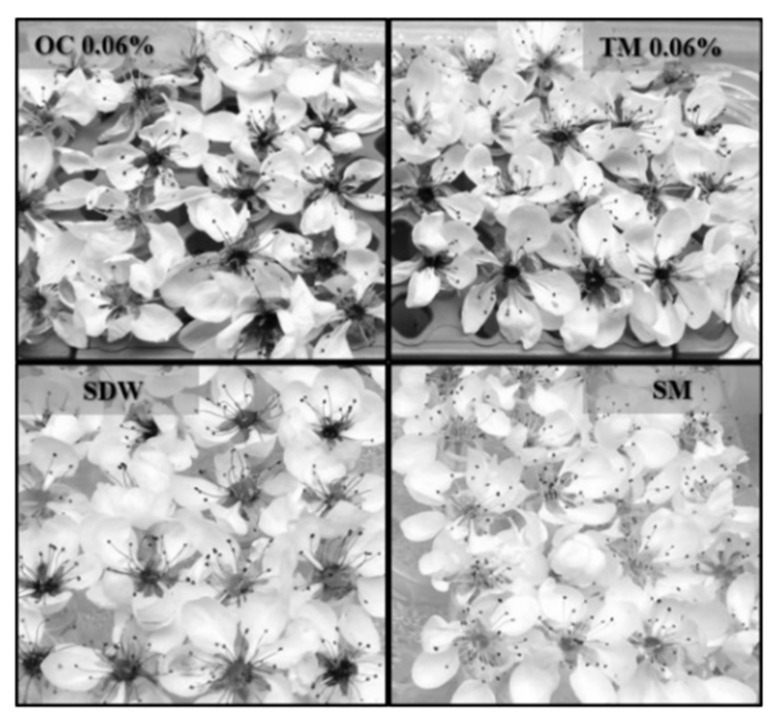
Fire blight incidence on pear flowers cv. Williams. Treatment: *O. compactum* CT carvacrol at 0.06% (OC 0.06%), *T. vulgaris* CT Thymol at 0.06% (TM 0.06%), streptomycin sulphate at 100 μg/mL (SM, positive control), sterile distilled water (SDW, negative control).

During the visual observation of symptoms on flowers, in the negative control, OC and TM, the necrosis of the calyx could be noted. On the contrary, in the flowers treated with SM, the calyx remained green (Figure 2).

Considering the negative results of the pear flowers experiment, in the ex vivo experiment on pear fruitlets cv. Abate Fétel, with the method of the fruitlets hole (Section 2.5.2), concentrations of 0.12% instead of 0.06% of EOs have been used.

**Figure 3:**
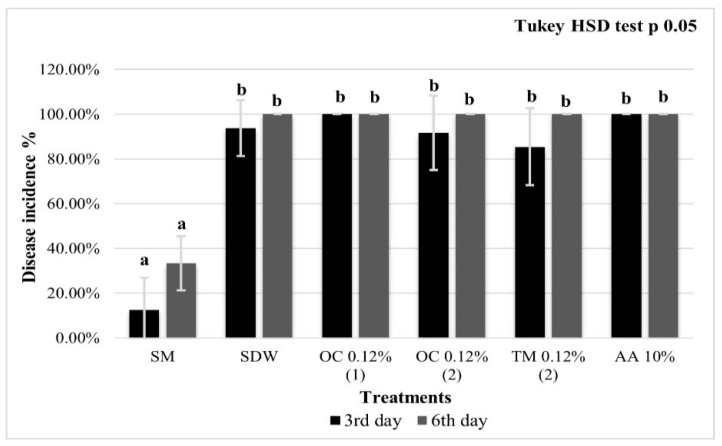
Holed pear fruitlets disease incidence. Immature pear fruits cv. Abate Fétel inoculated with *E. amylovora*, pre-treated with streptomycin sulphate at 100 μg/mL (SM, positive control); sterile distilled water (SDW, negative control); *O. compactum* CT carvacrol at 0.12% prepared in accordance with M1 (OC-M10.12%), *O. compactum* CT carvacrol at 0.12% prepared in accordance with M2 (OC-M2 0.12%), *T. vulgaris* CT thymol at 0.12% prepared in accordance with M2 (TM-M2 0.12%). Symptoms were recorded at 3 and 6 dpi. The bars represent standard deviation and the letters the statistical categories (Tukey HSD, *p* < 0.05).

The *E. amylovora* DI on immature fruitlets cv. Abate Fétel, inoculated and treated in the hole with OC and TM both at 0.12% prepared according to M1 and M2, and with AA at 10%, showed no significant effectiveness in reducing the disease incidence compared with negative control. After 3 dpi, the fruitlets treated with OC-M1 0.12% and with AA Hy (10%) showed 100% DI, similar to the negative control (approx. 90%). The incidence of fire blight on the pears treated with OC-M2 (0.12%) and TM-M2 (0.12%) was lower (approx. 87% and 82%, respectively) but not significantly different from the negative control. After 6 days, the DI was 100% in all treatments, except for the positive control (SM), which was approx. 12% and 32% after 3 and 6 dpi, respectively (Figure 3). On the third day after inoculation, M2 emulsions resulted in a DI of 92% and 85% for OC and TM, respectively, while the M1 emulsion showed an incidence of 100%, higher than the negative control (94%).

**Figure 4:**
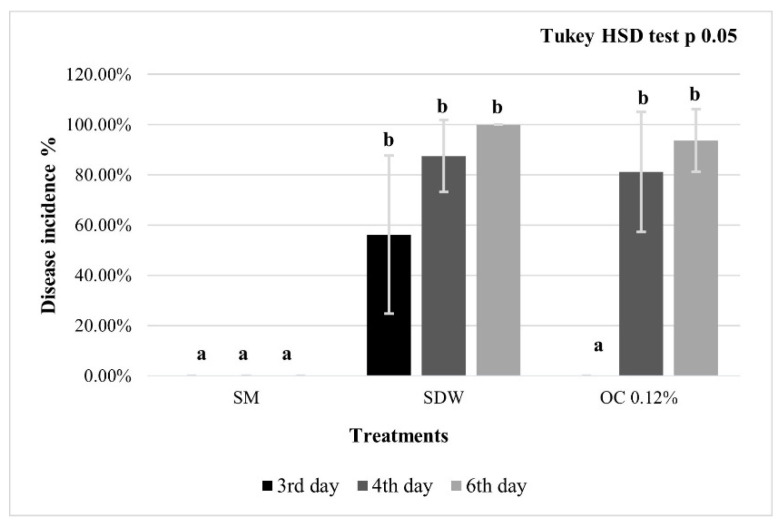
Punctured pear fruitlets disease incidence. Immature pears cv. Abate Fétel were pierced, inoculated with *E. amylovora*, and pre-treated with streptomycin sulphate at 100 μg/mL (SM, positive control), sterile distilled water (SDW, negative control); *O. compactum* at 0.12% prepared according to M2 (OC 0.12%). The observations were performed from 3 to 6 dpi. The bars indicate standard deviations, and the letters represent the statistical categories (Tukey HSD, *p* < 0.05).

In the second experiment on punctured pear fruitlets, at 3 dpi, the OC-M2 at 0.12% treatment was significantly effective as SM (positive control), whereas, at 4 and 6 dpi, there were no significant differences compared to the negative control (SDW; Figure 4).

### 3.4. Population Dynamics of Erwinia Amylovora on Treated Apple Flowers

**Figure 5:**
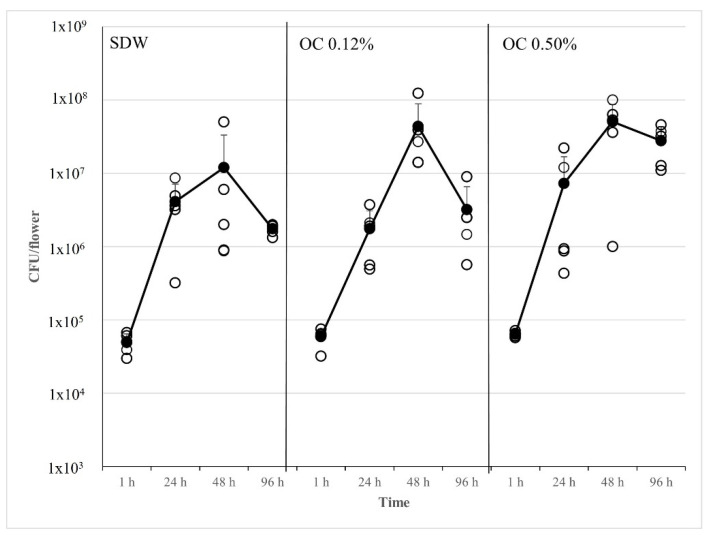
Population dynamics of *E. amylovora* rifampicin-resistant strain Ea 273R1, on apple blossoms cv. Rome Beauty pre-treated with sterile distilled water (SDW, negative control); *O. compactum* at 0.12% (OC 0.12%); *O. compactum* at 0.5% (OC 0.50%); the pathogen population was monitored for 96 h. Empty circles indicate the *E. amylovora* 273R1 strain population on each flower. The filled black circles and the black line indicate the mean population present in 5 flowers/day. Standard deviation bars are shown for each time point.

On apple flowers, the treatments OC at 0.12% and 0.5% did not reduce the population of the mutant strain *E. amylovora* 273R1 at each time point up to 96 h. At 1 h after pathogen inoculation, on flowers treated with OC at 0.12% and 0.5%, the *E. amylovora* mean population was comparable to that found on SDW (approx. 10^4^ CFU/flower); after 24 h and 48 h, the mean populations of *E. amylovora* increased, and the concentrations were similar in all treatments (approx. 10^6^ and 10^7^ CFU/flower, respectively). After 96 h, the pathogen population decreased to approx. 10^6^ CFU/flower on SDW and OC 0.12%, while it remained higher on flowers treated with OC 0.5% (approx. 10^7^ CFU/flower) (Figure 5). 

**Figure 6:**
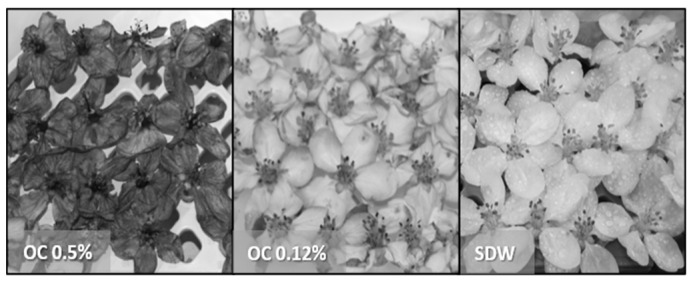
Apple blossoms, cv. Rome Beauty, treated to test population dynamics with EOs: *O. compactum* at 0.5%; note complete drying of the flowers (OC 0.5%), *O. compactum* at 0.12%; note slight darkening of the petals (OC 0.12%), sterile distilled water; note healthy flowers without darkening (SDW, negative control).

Both EO concentrations showed clear symptoms of phytotoxicity; the petals became brown after about 1 h, and later, the necrosis extended to the entire flower (Figure 6).

### 3.5. Scanning Electron Microscopy (SEM) Observations

**Figure 7:**
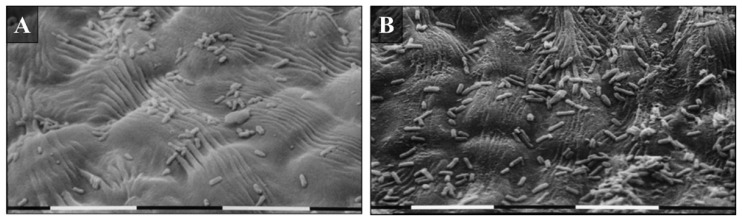
SEM observation of the *E. amylovora* 273R1 population, 48 h after its inoculation. Apple flowers hypanthium treated by nebulization of (**A**) sterile distilled water (negative control) and (**B**) *O. compactum* at 0.12% that shows a higher concentration of bacteria than negative treatment. The bars indicate the magnifications of 0.01 mm.

**Figure 8:**
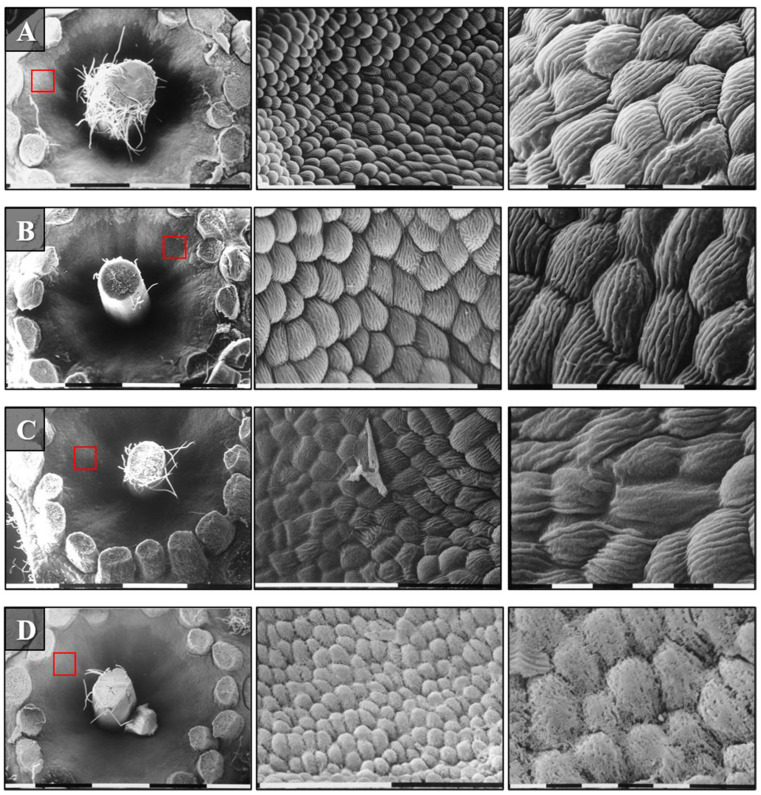
SEM observations of the apple flowers hypanthium surface treated and after 1 h sprayed with *E. amylovora* 273R1: (**A**) untreated and uninoculated flower; (**B**) flower treated with sterile distilled water (negative control) and inoculated; (**C**,**D**) flowers treated with *O. compactum* at 0.12% and 0.5% *v*/*v*, respectively, and inoculated. The red squares indicate the observation site. Bars indicate the magnifications and correspond to 1 mm, 0.1 mm, 0.01 mm, respectively.

**Figure 9:**
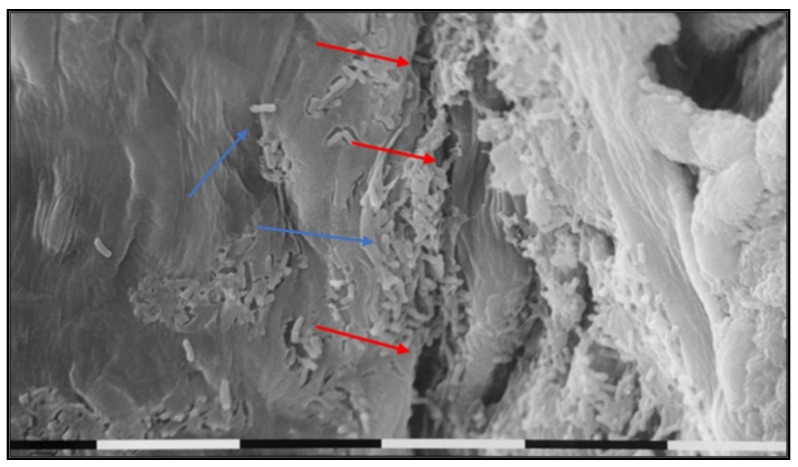
SEM observation of apple flower hypanthium surface after treatment with *O. compactum* at 0.5%, 48 h after inoculation with *E. amylovora* 273R1. The red arrows indicate tissue cracks caused by the treatment; blue arrows indicate bacterial cells. The bars indicate a magnification of 0.01 mm.

To confirm the results of the *E. amylovora* 273R1 mutant strain population dynamics (Figure 5), SEM observations were conducted. At 48 h after pathogen application, most cells resulted visible in the flowers treated with OC 0.12%, compared to the negative control (Figure 7). In addition, on the flowers treated with OC 0.5%, damages at the hypanthium were detected from 24 h after the treatment (Figure 8D and Figure 9). Figure 8 shows the different hypanthium tissue responses to all treatments, with evident cell degeneration in tissues treated with OC 0.5%. The untreated flowers and those treated with SDW remained intact until the last assessment at 96 h after pathogen inoculation or 120 h after their treatment (Figure 8).

### 3.6. Induced Resistance in Tomato Plants against Bacterial Leafspot

The two experiments on tomato plants cv. VF 10 under climatic chamber conditions showed a high disease pressure. Considering the possible phytotoxicity of EO, as described in the previous experiments, treatment with AA Hy has been added to this challenge. The rationale for the use of Hy is the lower concentration of active EO compounds in it, which, together with its higher solubility, minimizes phytotoxicity compared to potency.

**Figure 10:**
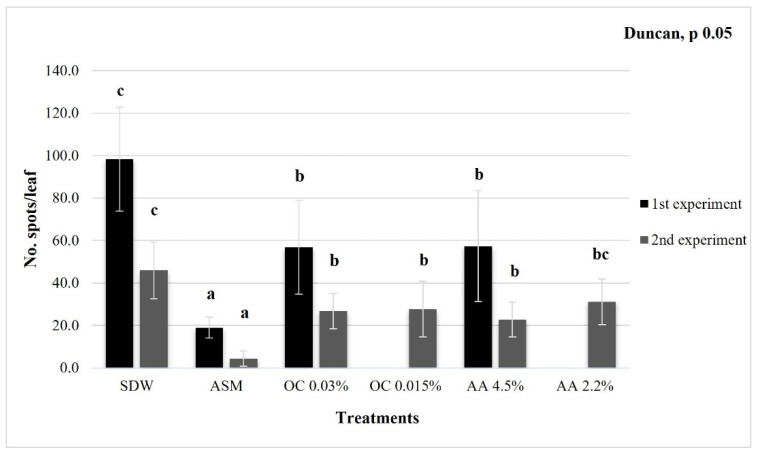
*In planta* experiments on bacterial leafspot control (*X. vesicatoria*) of tomato plants (cv. VF-10) pre-treated at the roots with sterile distilled water (SDW, negative control); acibenzolar-S-methyl (ASM, 75 µg/mL, positive control); *O. compactum* at 0.03% (OC 0.03%); *O. compactum* at 0.015% (OC 0.015%); *C. aurantium* var. *amara* Hy at 4.5% (AA 4.5%); *C. aurantium* var. *amara* Hy at 2.2% (AA 2.2%). The bars indicate standard deviations, and different letters show significant differences according to Duncan’s test (*p* < 0.05).

In the first experiment, the SDW-treated plants showed the highest disease severity (approx. 98 spots/leaf), significantly higher than that observed on plants treated with OC at 0.03% and AA at 4.5%, which were able to reduce the bacterial leafspot severity (approx. 57 spots/leaf in both treatments) (Figure 10).

In the second experiment, the disease severity was lower than in the first experiment. In the SDW-treated plants, the bacterial leafspot severity was the highest (approx. 46 spots/leaf) compared to all treatments. Tomato plants treated with AA at 4.5%, OC at 0.03% and OC at 0.015% showed significantly reduced disease severity (approx. 23, 27, and 28 spots/leaf, respectively) with respect to negative control; the treatment AA 2.2% was the least effective in reducing disease severity (approx. 31 spots/leaf) (Figure 10). The positive control treatment (ASM, acibenzolar-S-methyl 75 μg/mL) was able to significantly reduce BLST severity in both in planta experiments, with 19 and 4 spots/leaf in the first and second experiments, respectively (Figure 10).

## 4. Discussion

Microdilution tests have shown bacteriostatic and bactericidal activity for all tested OEs and Hys. The best MIC values (ranging from 0.015% to 0.06%) were recorded against *E. amylovora*, *X. vesicatoria*, and *A. vitis* by all OEs except TJ (ranging from 0.25% to 0.5%): these extremely low values of MIC evidenced the high antibacterial activity of the tested oils. On the other hand, *P. savastanoi* was the less sensitive strain to EOs, and this was probably due to the higher amount of saturated fatty acids linked to lipopolysaccharides in *Pseudomonas*’ outer membrane; this makes membranes less fluid and permeable, slowing down the passage of the active lipophilic molecules that characterize the antibacterial activity of EOs [32,33]. Similarly, the greater antibacterial efficacy of Hys against *P. savastanoi* could be explained by the hydrophilic nature of Hys, which might increase the rate of accumulation within the cell, making it more sensitive. Indeed, the MBC values of Hys, obtained for *P. savastanoi*, were equivalent to those of all the other strains studied.

The antibacterial activity of EOs, linked to their main constituents, such as carvacrol and thymol, is given by their chemical nature; in fact, phenolic monoterpenoids bind to plasma membranes through their hydroxyl group, causing a membrane fluidity decrease with a consequent loss of homeostasis, membrane depolarization, and, finally, cell death [34,35,36].

The HySTG’s low effectiveness, despite carvacrol being its main component, might be due to the lower quantity of active components present in the Hys with respect to an EO [2,3].

The *E. amylovora*–pomaceous organs pathosystem was chosen as the model for ex vivo direct inhibition tests, while the *Xanthomonas vesicatoria*–tomato pathosystem was used for indirect inhibition tests *in planta*; the macrodilution assays were carried out to verify the MBCs of OC, TM, and AA, which exhibited major activity versus *E. amylovora*, and the MBCs of OC and AA that have shown major activity versus Xv.

In the ex vivo DI experiment on flowers, although carvacrol and thymol are two isomers, with the same in vitro MIC values (0.06%) for both *O. compactum* CT carvacrol (OC) and *T. vulgaris* CT thymol (TM), the antimicrobial activity was slightly effective only in the OC 0.06% treatment after 4 dpi. Interestingly, at the same time, the treatment with OC 0.12% increased the DI. These results may be explained by the instability of emulsion, which might have caused the rapid volatilization of EO and uneven coverage of the treated surface. This can be due to the different sizes of the emulsion droplets, which tend to coalescence in an unstable emulsion, leading to different concentrations on the treated area [37,38,39,40]. This last factor may also explain the activity of OC 0.06% (DI approx. 42%) 4 dpi and the subsequent inefficacy at 6 dpi (DI 75%). Such treatment had a delaying effect in the expression of disease symptoms, but it did not reduce the fire blight incidence until the end of the trial. As previously noted, OC at 0.12% was the least effective treatment, at 4 days after inoculation. It seems possible that in the point where EOs drops were more concentrated damages in the hypanthium could be caused; these damages might have favored bacterial penetration into the tissue and a more intense bacterial growth as a consequence of the nutrients released from the flower wounds [41,42]. On the other hand, the same treatment, on the last day, was the most inhibiting (although not significant, DI approx. 71%), probably due to the major concentration, with respect to other treatments concentrations, which allowed larger EO amounts to remain in place longer, despite volatilization.

This EO “dose-dependent” mechanism could be explained by the cytotoxic effect that essential oils have at high concentrations compared to their beneficial effects at low concentrations [5,43]. To understand what most affected the result, another incidence test was carried out on immature pears, preparing the emulsion in two different ways. To verify if concentrations higher than MBC were cytotoxic for the plant rather than antibacterial, a population dynamic test of *E. amylovora* with a consequent observation of flowers at SEM was carried out.

In the first ex vivo test on holed pear fruitlets (first experiment), although there were no significant results, on the 3rd day a slight difference between the treatments obtained with the two emulsion methods was noted: on the 3rd day, the OC 0.12% emulsion prepared according to M1 showed a 100% DI, while OC and TM (both at 0.12%) emulsions prepared according to M2 showed a lower DI (approx. 90% and 85% respectively). This result may be explained by the instability of the emulsion: in fact, emulsion prepared on a heated magnetic stirrer is stable only immediately after preparation confirming the importance of the emulsion stability in in/ex vivo applications of EOs [38,39,40]. Furthermore, this finding agrees with the data obtained by Gutierrez et al. [44], which showed that the presence of starch negatively affects the efficacy of EOs, so the starch present in the unripe fruit pears might have influenced the EOs’ antibacterial activity. This negative interaction between starch and EOs have possibly been increased by the experimental method, for which EOs were placed into the hole made on the fruitlet’s surface, therefore in direct contact with the pulp containing the starch.

In the second experiment, on pierced pear fruitlets, the EO was not applied directly to the pulp, and the results obtained were significant only for the first day. The efficacy of the treatment on the first day could be since the EOs showed its antibacterial activity because they were not in direct contact with the pulp; on the other hand, in the following days, the efficacy of the treatment was reduced by the instability of the emulsion.

The population dynamics (PD) test and the subsequent SEM observations were carried out using the treatment with OC in two concentrations (0.12% and 0.5%, 2 and 8 times higher than MBC for *E. amylovora*, respectively) in order to verify if the OE ineffectiveness was due only to the emulsion’s instability or also to tissue damages. The PD data and SEM observations confirmed that tissue damage in flowers treated with 0.5% OC was detected as early as 1 h, as observed visually, supporting the hypothesis that in a complex system (in vivo and ex vivo), high doses of OEs become more harmful to eukaryotic cells [1,5,45]. Moreover, the low antibacterial efficacy of the treatments in ex vivo trials is most likely due to the intrinsic properties of OEs, such as the extreme volatility of some components, sensitivity to light and oxygen, and interactions in the phytocomplex [46,47]. The ineffectiveness of EOs related to their chemical nature could be overcome using different approaches such as polymers, emulsifiers, surfactants, solvents, or emulsion stabilizers, which make the emulsion particles very small and provide stability over time, uniform adhesion, and controlled release of the active principle [17,37,40,48]. In particular, nanoemulsions are characterized by greater kinetic and thermodynamic stability, by an easier diffusion during the application in addition to the transport of nanoparticles, greater incorporation, and the protection of hydrophilic or lipophilic molecules in dispersed phases; this facilitates the transport of bioactive molecules through plasma membranes, making the formulation more effective [17,40,48]. Moreover, the chelator ethylenediaminetetraacetic acid (EDTA), acting as a preservative agent in the *Calamintha officinalis* EO emulsion [49], could establish synergistic mechanisms with some EOs [50]. Furthermore, chitosan added to the emulsions creates a protective film that was effective in avoiding contact between oxygen and EOs, consequently prolonging their persistence [51,52,53].

Considering the direct ineffectiveness of EOs and Hys against *E. amylovora* on flowers and pear fruitlets, their indirect action as elicitors of defense responses mediated by the host was investigated on tomato plants against Xv. The *X. vesicatoria*–tomato pathosystem was chosen for its ease of use compared to an arboreous pathosystem. In both in planta experiments, the EO and Hy treatments applied at the roots were not in contact with the *X. vesicatoria* strain, which was inoculated at the leaves. In the first experiment, the plants treated with OC 0.03% and AA 4.5% showed a reduction in the bacterial spot severity (approx. 57 spot/leaf) in comparison to that evaluated on SDW-treated plants (approx. 98 spot/leaf). Both treatments provided a significant relative protection (approx. 42%), even if it was lower than the positive control (ASM), which provided approx. 81% relative protection [30]. Similar data were obtained in the second experiment assaying OC and AA at the above concentrations and half-diluted. The OC and AA seemed to elicit a host defense response towards Xv. The disease pressure was higher in the first experiment, where the plants treated at the roots with SDW showed the highest disease severity (approx. 98 spots/leaf), while in the second experiment, the bacterial leafspot severity was lower (approx. 46 spots/leaf). Moreover, in both experiments, the relative protection induced by 0.03% OC and 4.5% AA ranged between approx. 42 and 50%. Finally, in the second experiment, the treatments with 0.015% OC and 2.2% AA provided relative protection of 40% and 32%, respectively; in the plants treated with 2.2% AA, the disease severity was only slightly reduced with respect to the negative control (SDW).

The induction of EO resistance is not fully explained in the literature; some studies evaluated, in addition to disease reduction (against both fungal and bacterial pathogens), the expression increases in antioxidant enzymes and/or chitinases and β-glucanase in response to EOs alone or polysaccharides associated, demonstrating that essential oil has an activity directly on the plant and indirectly on pathogens [54,55]. Banani et al. [56] in their ex vivo experiments, showed the reduction of fungal pathogen growth and an increase of PR8 transcripts (“Pathogenesis-Related protein”, PR), in response to treatments with thyme EO on apple fruits.

A more in-depth study has been conducted by Rienth et al. [57]; the study provides information regarding the underlying mechanisms in the host–pathogen EO interaction and clearly shows that *O. vulgare* EO treatment triggered a multilayered immune system in plants. In particular, the transcriptome analysis of grapevines, treated against *Plasmopara viticola* by fumigation with oregano EO, evaluated the activation of the plant immune system through the transcription increase in genes involved in the salicylic acid, jasmonic acid, and ethylene pathways; the transcription of PR protein genes; and through the synthesis of phytoalexins.

However, further enzymatic or transcriptomic analyses will be required to confirm the ability of EOs and Hys to induce a defense response in tomato plants against *X. vesicatoria* infections.

To sum up, this paper highlights the potentiality, but also the weakness, of EOs and Hys versus several bacterial pathogens. With opportune further investigations, botanicals could become precious allies in sustainable disease control in agriculture.

## 5. Conclusions

All the EOs in this study (especially those with the carvacrol chemotype) proved to be active against several phytopathogenic bacterial species in vitro, but their ex vivo efficacy remains strongly influenced by the emulsion stability. Both EOs and Hys are promising tools for sustainable defense, but its formulation (for example, through nanoemulsions, microencapsulations, etc.) is a crucial step to enable them to enhance both their biological activity and stability over an extended period. In this study, it was seen that the antibacterial ex vivo activity of EOs is strongly related to concentration: doses higher than MBC, directly applied, become cytotoxic even for the host, while doses equal or sub-MBC used as resistance inducers can exert an inhibitory action on the bacterium without damaging the plant. This finding could create important scenarios for the use of these products as inducers. It will be useful in the future to investigate this property following Hy irrigation, a more practical method of application. Medicinal plant producers involved in the extraction of EOs often accumulate copious quantities of Hys due to their low market demand. Irrigation with hydrolates could be useful for producers to prevent the establishment of crop diseases but also for the use of surplus Hys in a circular economy perspective. In addition, it could be interesting to assess the metabolic effect of Hy and EO treatments on the plants, as it could be doubly useful if it increased the production of secondary metabolites, making the officinal crops potentially richer in EOs. Another application of Hys worth exploring could be aspersion, which could be useful in controlling the inoculum of those pathogens commonly coexisting in the field (e.g., *P. savastanoi* pv. *savastanoi*, the causal agent of the olive knot), as well as in the post-harvest treatment. In conclusion, both essential oils and hydrolates are promising tools for sustainable defence, but it would be desirable, before their probable spread in agriculture, to keep in mind that for their physiological and complex origin from the plants, they can play diversified actions in and outside of the plant as communication signals with the surrounding environment. Further studies on the mechanism of action not only against microorganisms but especially within the plant will be needed for their safe and promising use in agriculture.

## Figures and Tables

**Table 1:** Treatments and emulsion type methods used in the first experiment on immature pear fruitlets under climatic chamber conditions.

Treatment	Emulsion Method
Sterile distilled water (SDW)	
OC 0.12%	M1
OC 0.12%	M2
TM 0.12%	M2
AA ^1^ 10%	
Streptomycin sulphate (SM) 100 ppm	

^1^ Hydrolates did not need emulsion.

**Table 2:** Partial chemical composition of the essential oils (EOs) tested. Only the major constituents for each EO are reported.

Essential Oils	Main Components	Relative Area (%)
*T. vulgaris* CT thymol	Thymol	46.84
	p-Cymene	15.61
	Υ-Terpinene	9.18
	Linalool	4.63
	Carvacrol	4.58
	β-Caryophyllene	2.64
*T. vulgaris* CT thujanol	Trans-Thujanol	22.71
	Terpinene-4-OL	12.05
	Linalool	9.45
	Myrcenol	7.90
	Υ-Terpinene	5.93
	Cis-Thujanol	4.73
	β-Myrcene	4.41
	Myrcenyl acetate	3.96
	α-Terpinene	3.30
	Limonene	2.70
	α-Terpineol	2.62
*O. compactum* CT carvacrol	Carvacrol	48.13
	Thymol	14.15
	Υ-Terpinene	13.16
	p-Cymene	11.05
*S. montana* CT carvacrol	Carvacrol	63.16
	p-Cymene	9.82
	Υ-Terpinene	1.44
	α-Terpinene	1.98

**Table 3:** Partial chemical composition of the hydrolates (Hys) tested. Only the major constituents for each EO are reported.

Hydrolates	Main Components	Relative Area (%)
*C. aurantium* var. *amara*	Linalool	47.7
	Terpinolene	24.82
	α-Terpineol	13.83
	Geraniol	6.43
	Carvone	2.14
	Citronellol	2.14
	Nerol	1.93
*S. montana*	Carvacrol	87.79
	Thymol	13.88

**Table 4:** Minimum inhibitory (MIC) and bactericidal concentration (MBC) values of EOs and Hys tested against phytopathogenic bacterial strains. The MIC and MBC concentrations are expressed in percentage (% *v*/*v*); in bold, the lower MBC values for each strain.

	Bacteria							
	*Erwinia amylovora*		*Pseudomonas savastanoi* ssp. *savastanoi*		*Xanthomonas vesicatoria*		*Allorhizobium vitis*	
Essential Oils	MIC	MBC	MIC	MBC	MIC	MBC	MIC	MBC
*Origanum compactum* (CT carvacrol) (OC)	0.03	**0.06**	0.03	**0.125**	0.03	**0.03**	0.015	**0.03**
*Thymus vulgaris* (CT thymol) (TM)	0.03	**0.06**	0.125	0.5	0.03	0.06	0.015	**0.03**
*Thymus vulgaris* (CT thujanol) (TJ)	0.25	0.5	0.5	1	0.25	0.25	0.5	0.125
*Satureja montana* (CT carvacrol)	0.06	**0.06**	0.06	0.2	0.03	**0.03**	0.03	**0.03**
**Hydrolates**								
*Satureja montana* (HySTG)	25	50	50	50	25	50	12.5	50
*Citrus aurantium* var. *amara* (AA)	6.25	**6.25**	1.6	**3.1**	3.1	**3.1**	0.8	**3.1**

## Data Availability

Not applicable.

## Supplementary Materials

The following supporting information can be downloaded at: www.mdpi.com/article/10.3390/microorganisms10040702/s1, Figure S1: *Erwinia amylovora* inhibition test by macrodilution, Figure S2: *Xanthomonas vesicatoria* inhibition test by macrodilution, Table S1: *Origanum compactum* GCMS analysis, Table S2: *Satureja montana* GCMS analysis, Table S3: *Thymus vulgaris* (CT thymol) GCMS analysis, Table S4: *Thymus vulgaris* (CT thujanol) GCMS analysis, Table S5: *Satureja montana* hydrolate GCMS analysis, Table S6: *Citrus aurantium* var. *amara* hydrolate GCMS analysis.

## References

[1] Bakkali F., Averbeck S., Averbeck D., Idaomar M. (2008). Biological Effects of Essential Oils—A Review. Food Chem. Toxicol..

[2] Labadie C., Ginies C., Guinebretiere M.H., Renard C.M.G.C., Cerutti C., Carlin F. (2015). Hydrosols of Orange Blossom (*Citrus aurantium*), and Rose Flower (*Rosa damascena* and *Rosa centifolia*) Support the Growth of a Heterogeneous Spoilage Microbiota. Food Res. Int..

[3] Di Vito M., Bellardi M.G., Mondello F., Modesto M., Michelozzi M., Bugli F., Sanguinetti M., Sclocchi M.C., Sebastiani M.L., Biffi S. (2019). Monarda Citriodora Hydrolate vs Essential Oil Comparison in Several Anti-Microbial Applications. Ind. Crops Prod..

[4] Zitzelsberger C., Buchbauer G. (2015). Essential Oils as “A Cry for Help”. A Review. Nat. Prod. Commun..

[5] Sharifi-Rad J., Sureda A., Tenore G.C., Daglia M., Sharifi-Rad M., Valussi M., Tundis R., Sharifi-Rad M., Loizzo M.R., Oluwaseun Ademiluyi A. (2017). Biological Activities of Essential Oils: From Plant Chemoecology to Traditional Healing Systems. Molecules.

[6] Choudhary P., Aggarwal P.R., Rana S., Nagarathnam R., Muthamilarasan M. (2021). Molecular and Metabolomic Interventions for Identifying Potential Bioactive Molecules to Mitigate Diseases and Their Impacts on Crop Plants. Physiol. Mol. Plant Pathol..

[7] Hyldgaard M., Mygind T., Meyer R.L. (2012). Essential Oils in Food Preservation: Mode of Action, Synergies, and Interactions with Food Matrix Components. Front. Microbiol..

[8] Carvalho I.T., Estevinho B.N., Santos L. (2016). Application of Microencapsulated Essential Oils in Cosmetic and Personal Healthcare Products—A Review. Int. J. Cosmet. Sci..

[9] Pandey A.K., Kumar P., Singh P., Tripathi N.N., Bajpai V.K. (2017). Essential Oils: Sources of Antimicrobials and Food Preservatives. Front. Microbiol..

[10] Yang S.K., Low L.Y., Yap P.S.X., Yusoff K., Mai C.W., Lai K.S., Lim S.H.E. (2018). Plant-Derived Antimicrobials: Insights into Mitigation of Antimicrobial Resistance. Rec. Nat. Prod..

[11] Aljaafari M.N., Alali A.O., Baqais L., Alqubaisy M., Alali M., Molouki A., Ong-Abdullah J., Abushelaibi A., Lai K.S., Lim S.H.E. (2021). An Overview of the Potential Therapeutic Applications of Essential Oils. Molecules.

[12] European Commission (2002). Commission Regulation (EC) No. 473/2002 of 15 March 2002 Amending Annexes I, II and VI to Council Regulation (EEC) No. 2092/91 on Organic Production of Agricultural Products and Indications Referring Thereto on Agricultural Products and Foodstuffs, and Laying down Detailed Rules as Regards the Transmission of Information on the Use of Copper Compounds.

[13] Lamichhane J.R., Osdaghi E., Behlau F., Köhl J., Jones J.B., Aubertot J.N. (2018). Thirteen Decades of Antimicrobial Copper Compounds Applied in Agriculture. A Review. Agron. Sustain. Dev..

[14] Goto M., Hikota T., Nakajima M., Takikawa Y., Tsuyumu S. (1994). Occurrence and Properties of Copper-Resistance in Plant Pathogenic Bacteria. Ann. Phytopath. Soc. Jpn..

[15] Cameron A., Sarojini V. (2014). *Pseudomonas syringae* pv. *actinidiae*: Chemical Control, Resistance Mechanisms and Possible Alternatives. Plant Pathol..

[16] Oliveira da Silva É., Martins S.J., Alves E. (2014). Essential Oils for the Control of Bacterial Speck in Tomato Crop. Afr. J. Agric. Res..

[17] Moghimi R., Ghaderi L., Rafati H., Aliahmadi A., Mcclements D.J. (2016). Superior Antibacterial Activity of Nanoemulsion of Thymus Daenensis Essential Oil against *E. Coli*. Food Chem..

[18] Mattarelli P., Epifano F., Minardi P., Di Vito M., Modesto M., Barbanti L., Bellardi M.G. (2017). Chemical Composition and Antimicrobial Activity of Essential Oils from Aerial Parts of Monarda Didyma and Monarda Fistulosa Cultivated in Italy. J. Essent. Oil Bear. Plants.

[19] Orzali L., Valente M.T., Scala V., Loreti S., Pucci N. (2020). Antibacterial Activity of Essential Oils and Trametes Versicolor Extract against Clavibacter Michiganensis Subsp. Michiganensis and Ralstonia Solanacearum for Seed Treatment and Development of a Rapid in Vivo Assay. Antibiotics.

[20] Di Vito M., Bellardi M.G., Colaizzi P., Ruggiero D., Mazzuca C., Micheli L., Sotgiu S., Iannuccelli S., Michelozzi M., Mondello F. (2018). Hydrolates and Gellan: An Eco-Innovative Synergy for Safe Cleaning of Paper Artworks. Stud. Conserv..

[21] King E.O., Ward M.K., Raney D.E. (1954). Two Simple Media for the Demonstration of Pyocyanin and Fluorescin. J. Lab. Clin. Med..

[22] Dye M.H., Clarke G., Wain R.L. (1962). Investigations on the Auxins in Tomato Crown-Gall Tissue. Proc. R. Soc. Lond. Ser. B Biol. Sci..

[23] Biondi E., Bini F., Anaclerio F., Bazzi C. (2009). Effect of Bioagents and Resistance Inducers on Grapevine Crown Gall. Phytopathol. Mediterr..

[24] (2003). European Committee on Antimicrobial Susceptibility Testing (EUCAST); European Committee for Antimicrobial Susceptibility Testing of the European Society of Clinical Microbiology and Infectious Diseases (ESCMID). Clin. Microbiol. Infect..

[25] Wald-Dickler N., Holtom P., Spellberg B. (2018). Busting the Myth of “Static vs Cidal”: A Systemic Literature Review. Clin. Infect. Dis. Off. Publ. Infect. Dis. Soc. Am..

[26] Pusey P.L. (1997). Crab Apple Blossoms as a Model for Research on Biological Control of Fire Blight. Phytopathology.

[27] (2013). European and Mediterranean Plant Protection Organization (OEPP/EPPO) PM 7/20 (2) Erwinia Amylovora. EPPO Bull..

[28] Minardi P., Mucini S., Mazzucchi U. (2012). Molecular Analysis of the DspEF Locus in Erwinia Amylovora Wild Strains of Northern Italy. J. Plant Pathol..

[29] Biondi E., Bazzi C., Vanneste J.L. (2006). Reduction of Fire Blight Incidence on Apple Flowers and Colonisation of Pear Shoots in Experimental Orchards Using Pseudomonas spp. IPV-BO G19 and IPV-BO 3371. Acta Hortic..

[30] Perez S.M., Biondi E., Laurita R., Proto M., Sarti F., Gherardi M., Bertaccini A., Colombo V. (2019). Plasma Activated Water as Resistance Inducer against Bacterial Leaf Spot of Tomato. PLoS ONE.

[31] Koenraadt H., van Betteray B., Germain R., Hiddink G., Jones J.B., Oosterhof J., Rijlaarsdam A., Roorda P., Woudt B. (2009). Development of Specific Primers for the Molecular Detection of Bacterial Spot of Pepper and Tomato. Acta Hortic..

[32] Nikaido H. (1994). Prevention of Drug Access to Bacterial Targets: Permeability Barriers and Active Efflux. Science.

[33] Kalemba D., Kunicka A. (2003). Antibacterial and Antifungal Properties of Essential Oils. Curr. Med. Chem..

[34] Helander I.M., Alakomi H.L., Latva-Kala K., Mattila-Sandholm T., Pol I., Smid E.J., Gorris L.G.M., von Wright A. (1998). Characterization of the Action of Selected Essential Oil Components on Gram-Negative Bacteria. J. Agric. Food Chem..

[35] Lambert R.J.W., Skandamis P.N., Coote P.J., Nychas G.-J.E. (2001). A Study of the Minimum Inhibitory Concentration and Mode of Action of Oregano Essential Oil, Thymol and Carvacrol. J. Appl. Microbiol..

[36] Rempe C.S., Burris K.P., Lenaghan S.C., Stewart C.N. (2017). The Potential of Systems Biology to Discover Antibacterial Mechanisms of Plant Phenolics. Front. Microbiol..

[37] ICI Americas Inc. (1980). The HLB SYSTEM a Time-Saving Guide to Emulsifier Selection.

[38] Fernandes C.P., Mascarenhas M.P., Zibetti F.M., Lima B.G., Oliveira R.P.R.F., Rocha L., Falcão D.Q. (2013). HLB Value, an Important Parameter for the Development of Essential Oil Phytopharmaceuticals. Rev. Bras. Farmacogn..

[39] Ghosh V., Mukherjee A., Chandrasekaran N. (2013). Ultrasonic Emulsification of Food-Grade Nanoemulsion Formulation and Evaluation of Its Bactericidal Activity. Ultrason. Sonochem..

[40] Borges D.F., Lopes E.A., Fialho Moraes A.R., Soares M.S., Visôtto L.E., Oliveira C.R., Moreira Valente V.M. (2018). Formulation of Botanicals for the Control of Plant-Pathogens: A Review. Crop Prot..

[41] Bubán T., Orosz-Kovács Z., Farkas Á. (2003). The Nectary as the Primary Site of Infection by Erwinia Amylovora (Burr.) Winslow et al.: A Mini Review. Plant Syst. Evol..

[42] Farkas Á., Orosz-Kovács Z., Déri H., Chauhan S.V.S. (2007). Floral Nectaries in Some Apple and Pear Cultivars with Special Reference to Bacterial Fire Blight. Curr. Sci..

[43] Efferth T., Koch E. (2011). Complex Interactions between Phytochemicals. The Multi-Target Therapeutic Concept of Phytotherapy. Curr. Drug Targets.

[44] Gutierrez J., Barry-Ryan C., Bourke P. (2008). The Antimicrobial Efficacy of Plant Essential Oil Combinations and Interactions with Food Ingredients. Int. J. Food Microbiol..

[45] Saad N.Y., Muller C.D., Lobstein A. (2013). Major Bioactivities and Mechanism of Action of Essential Oils and Their Components. Flavour Fragr. J..

[46] Acedo-Carrillo J.I., Rosas-Durazo A., Herrera-Urbina R., Rinaudo M., Goycoolea F.M., Valdez M.A. (2006). Zeta Potential and Drop Growth of Oil in Water Emulsions Stabilized with Mesquite Gum. Carbohydr. Polym..

[47] Turek C., Stintzing F.C. (2012). Impact of Different Storage Conditions on the Quality of Selected Essential Oils. Food Res. Int..

[48] Elmer W., White J.C. (2018). The Future of Nanotechnology in Plant Pathology. Annu. Rev. Phytopathol..

[49] Nostro A., Cannatelli M.A., Morelli I., Cioni P.L., Bader A., Marino A., Alonzo V. (2002). Preservative Properties of Calamintha Officinalis Essential Oil with and without EDTA. Lett. Appl. Microbiol..

[50] Skandamis P., Koutsoumanis K., Fasseas K., Nychas G.-J.E. (2001). Inhibition of Oregano Essential Oil and EDTA on Escherichia Coli O157:H7. Ital. J. Food Sci..

[51] Hosseini M.H., Razavi S.H., Mousavi S.M.A., Yasaghi S., Ahmad S., Hasansaraei A.G. (2008). Improving Antibacterial Activity of Edible Films B. J. Appl. Sci..

[52] Bonilla J., Atarés L., Vargas M., Chiralt A. (2012). Effect of Essential Oils and Homogenization Conditions on Properties of Chitosan-Based Films. Food Hydrocoll..

[53] Grande-Tovar C.D., Serio A., Delgado-Ospina J., Paparella A., Rossi C., Chaves-López C. (2018). Chitosan Films Incorporated with Thymus Capitatus Essential Oil: Mechanical Properties and Antimicrobial Activity against Degradative Bacterial Species Isolated from Tuna (*Thunnus* sp.) and Swordfish (*Xiphias gladius*). J. Food Sci. Technol..

[54] Bill M., Sivakumar D., Korsten L., Thompson A.K. (2014). The Efficacy of Combined Application of Edible Coatings and Thyme Oil in Inducing Resistance Components in Avocado (*Persea americana* Mill.) against Anthracnose during Post-Harvest Storage. Crop Prot..

[55] Luiz C., da Rocha Neto A.C., Franco P.O., di Piero R.M. (2017). Emulsions of Essential Oils and Aloe Polysaccharides: Antimicrobial Activity and Resistance Inducer Potential against Xanthomonas Fragariae. Trop. Plant Pathol..

[56] Banani H., Olivieri L., Santoro K., Garibaldi A., Gullino M.L., Spadaro D. (2018). Thyme and Savory Essential Oil Efficacy and Induction of Resistance against Botrytis Cinerea through Priming of Defense Responses in Apple. Foods.

[57] Rienth M., Crovadore J., Ghaffari S., Lefort F. (2019). Oregano Essential Oil Vapour Prevents Plasmopara Viticola Infection in Grapevine (*Vitis vinifera*) and Primes Plant Immunity Mechanisms. PLoS ONE.

