# Supplementary material for: Essential Oils and Hydrolates: Potential Tools for Defense against Bacterial Plant Pathogens
Source: Microorganisms. 2022 Mar 24;10(4):702. doi: 10.3390/microorganisms10040702 (PMC9031397; doi:10.3390/microorganisms10040702)
Supplement: Supplementary file 1 [file microorganisms-10-00702-s001.zip › microorganisms-1630647-supplementary.pdf]

Supplementary file

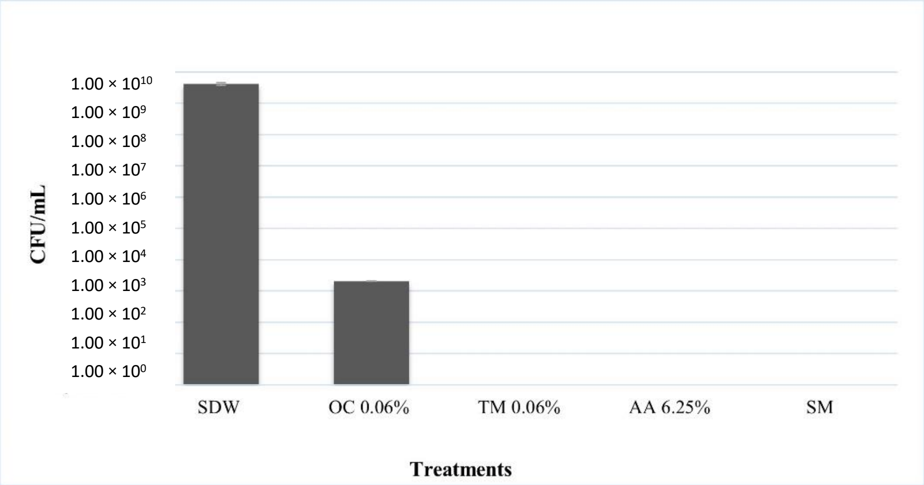

Figure S1

Erwinia amylovora (Ea) inhibition test by macrodilution, after 24 h of incubation. SDW, sterile distilled water as negative control; OC, *O. compactum* (CT carvacrol) Essential Oil; TM, *T. vulgaris* CT thymol Essential Oil; AA, *C. aurantium* var. *amara* Hydrolate; SM, Streptomycin (100 µg/mL) as a positive control. In X-axis MBC values expressed in percentage are shown. The bars indicate standard deviations.

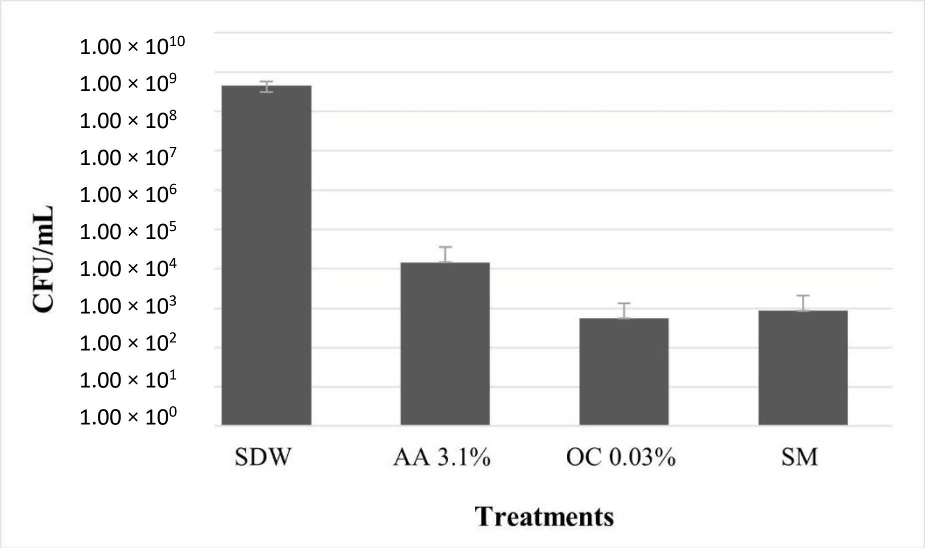

Figure S2

Xanthomonas vesicatoria (Xv) inhibition test by macrodilution, after 24 h of incubation. SDW, sterile distilled water as negative control; OC, *O. compactum* (CT carvacrol) EO; AA, *C. aurantium* var. *amara* hydrolate;

SM, Streptomycin (100 μg/mL) as positive control. In X-axis MBC values expressed in percentage are shown. The bars indicate standard deviations.

Table S1 *Origanum compactum* (CT carvacrol) GCMS analysis.

RT	Costituents	Percentage
4.8	Ethyl formate	0.03
5.1	2-Methyl Butanal	0.01
5.2	Isovaleraldehyde	0.03
5.6	2-Ethyl Furane	0.01
6.6	Tricyclene	0.01
6.8	Methyl Isovalerate	0.03
7.0	α-Pinene	0.69
7.1	α-Thujene	0.76
8.0	Ethyl Isovalerate	0.01
8.1	Camphene	0.12
9.4	β-Pinene	0.13
10.1	4-Methyl-3-Penten-2-one	0.04
10.9	Δ3-Carene	0.08
11.0	3-Heptanon	0.01
11.5	β-Myrcene	1.57
11.6	α-Phellandrene	0.20
11.8	ψ-Limonene	0.01
12.2	α-Terpinene	1.67
12.6	2.3-Dehydro-1.8-Cineole	0.02
13.1	Lcimonene	0.26
13.6	1.8-Cineole + β-Phellandrene	0.32
13.9	2-Hexenal	0.01
14.5	Cis-β-Ocimene	0.02
15.6	**γ-Terpinene**	**13.16**
15.7	Trans-β-Ocimene	0.07
15.9	3-Octanon	0.12
16.8	**p-Cymene**	**11.05**
17.4	Terpinolene	0.10
21.8	4-Hydroxy-4-Methyl-2-Pentanone	0.02
23.1	3-Hexen-1-ol	0.01
23.9	Menthatrien Isomer	0.01
24.0	3-Octanol	0.02
25.7	α-Thujone	0.01
26.1	Terpenic Cetone	0.04
26.5	α.p-Dimethylstyrene	0.13
26.7	Menthatriene Isomer	0.06
26.8	Linalool cis-Oxide	0.01
27.4	1-Octen-3-ol	0.20
27.8	Furfural	0.01
28.4	Trans-Thujanol	0.12
28.6	Linalool trans-Oxide	0.02

31.2	Camphor	0.08
31.3	Terpenic CetoneMw=152	0.05
31.6	Terpenic CetoneMw=152	0.06
32.5	α-Gurjuyene	0.01
33.6	Linalool	1.36
33.7	Cis-Thujanol	0.06
33.7	1-Nonen-3-ol	0.03
34.1	1-Octanol	0.01
34.4	Trans-P-Menth-2-en-1-ol	0.03
36.6	β-Caryophyllene	1.60
36.8	Terpinene-4-ol	0.67
37.1	Sesquiterpene	0.04
37.2	Methyl Carvacrol Ether	0.08
37.3	Hotrienol	0.02
38.2	Trans-Dihydrocarvon	0.04
38.3	Cis-p-Menth-2-en-1-Ol	0.02
39.2	Pulegone	0.02
39.5	Allo-Aromadendrene	0.03
40.4	1-Nonanol	0.04
40.8	α-Humulene	0.10
41.4	Compound Mw=152	0.02
41.8	Neral	0.10
42.1	Carvotanacetone	0.01
42.5	α-Terpineol	0.32
42.6	Borneol	0.34
43.5	Carvenone	0.02
43.9	Terpenic compound Mw=152	0.02
44.1	Menthadienol Isomer	0.02
44.2	α-Muurolene	0.02
44.4	β-Bisabolene	0.05
44.5	Carvone	0.03
44.8	Terpenic compound Mw=152	0.02
45.3	1-Decanol	0.01
46.0	δ-Cadinene	0.09
46.1	γ-Cadinene	0.04
46.5	Methylacetophenon	0.01
47.1	Cuminal	0.02
47.3	Menthadienol Isomer	0.01
48.6	Verbenyl Acetate	0.06
49.8	Sabinol Isomer	0.01
50.1	Trans-Carveol	0.03
50.9	P-Cymene-8-ol	0.13
54.7	Isopropyl Phenotole	0.05
58.1	Isocaryophyllene Oxide	0.02
58.5	Caryophyllene Oxide	0.16
64.1	Cuminol	0.05
65.7	Spathulenol	0.02
67.5	Isothymol	0.09

68.3	**Thymol**	**14.15**
69.1	Isocarvacrol	0.12
69.9	**Carvacrol**	**48.13**
70.7	Sesquiterpenol	0.03
71.5	Sesquiterpenol	0.02
73.2	Vinyl-Quajacol	0.06
73.7	Caryophylla-3.7-dien-6-ol	0.02
75.5	Sesquiterpenol	0.02
77.3	Sesquiterpenic epoxide	0.02
79.1	Phenolic compound	0.02
104.5	Hydroxymethoxyphenyl Ethanone	0.03
	**TOTAL**	**99.94**

Table S2 *Satureja montana* (CT carvacrol) GCMS analysis

**RT**	**Costituents**	**Percentage**
5.082	2.3-Dimethyl-3-buten-2-ol	0.05
6.717	Tricyclene	0.01
6.872	β-Thujene	1.11
7.096	α-Pinene	0.76
7.464	Dehydrosabinene	0.01
7.599	Camphene	0.22
8.484	Sabinene	0.07
8.599	β-Pinene	0.11
8.672	1-Octen-3-ol	0.47
9.136	β-Myrcene	0.95
9.655	α-Phellandrene	0.21
9.886	3-Carene	0.06
10.138	α-Terpinene	1.98
10.45	p-Cymene	9.82
10.63	Limonene	0.66
10.713	Eucalyptol	0.20
11.05	Cis-β-Ocimene	0.02
11.478	Trans-β-Ocimene	0.04
11.891	**γ-Terpinene**	**13.44**
12.233	No Match	0.55
13.175	Terpinolene	0.05
13.589	Cis-Sabinene Hydrate	0.07
13.68	β-Linalool	0.48
16.57	Borneol	0.50
17.108	L-Terpinen-4-ol	0.29
17.498	p-Cymen-8-ol	0.01
17.729	α-Terpineol	0.06
20.105	1.3.5.5.6.6-Hexamethyl-1.3-Cyclohexadiene	0.08
20.346	Tymoquinone	0.03
22.289	Thymol	1.21
22.715	**Carvacrol**	**63.16**
24.186	δ-Elemene	0.06
25.704	Carvacrol acetate	0.13

25.839	α-Copaene	0.05
26.214	β-Bourbonene	0.04
26.535	β-Elemene	0.01
27.644	β-Caryophyllene	1.53
28.051	β-Cubebene	0.04
29.04	Humulene	0.03
29.991	γ-Cadinene	0.02
30.168	Germacrene D	0.28
30.79	γ-Elemene	0.09
31.306	β-Bisabolene	0.88
31.484	γ-Cadinene	0.02
31.86	δ-Cadinene	0.07
34.205	Caryophyllene oxide	0.07
	**TOTAL**	**100**

Table S3: *Thymus vulgaris* (CT thymol) GCMS analysis

RT	Costituents	Percentage
4.7	2-Methyl Butanal	0.01
4.8	Isovaleraldehyde	0.02
6.0	2-Methylbutyrate	0.01
6.1	Tricyclene	0.09
6.3	α-Pinene	0.94
6.4	α-Thujene	1.11
6.8	β-Fenchene	0.01
7.1	α-Fenchene	0.04
7.3	Camphene	1.37
8.4	β-Pinene	0.25
8.8	Sabinene	0.03
8.9	Pinadiene	0.02
9.1	Thujadiene	0.02
9.7	Δ3-Carene	0.07
9.8	3-Heptanon	0.01
10.2	β-Myrcene	1.67
10.3	α-Phellandrene	0.19
10.5	ψ-Limonene	0.01
10.9	α-Terpinene	1.46
11.7	Limonene	0.48
12.1	1.8-Cineole	0.15
12.2	β-Phellandrene	0.43
13.2	Cis-β-Ocimene	0.02
13.9	**γ-Terpinene**	**9.18**
14.1	Trans-β-Ocimene	0.09
14.2	3-Octanone	0.04
14.5	Aliphatic Ester	0.01
15.1	**p-Cymene**	**15.61**
15.6	Terpinolene	0.13
18.6	6-Methyl-5-Hepten-2-one	0.01
18.8	Prenyl Isobutyrate	0.02
19.4	1-Hexanol	0.02
19.6	Aliphatic Ester	0.02
21.2	3-Hexen-1-ol	0.01

21.9	Aliphatic Ester	0.01
22.0	3-Octanol	0.03
24.1	Terpenic Cetone	0.03
24.5	α.p-Dimethylstyrene	0.05
24.7	Linalool cis-Oxide	0.03
25.3	1-Octen-3-ol	0.06
25.8	Furfural	0.01
26.0	Sesquiterpene	0.01
26.2	Trans-Thujanol	0.23
26.5	Linalool trans-oxide	0.05
27.2	Isogeranial	0.03
27.8	α-Copaene	0.03
28.8	Isoneral	0.03
29.1	Camphor	0.97
29.4	β-Bourbonene	0.01
30.1	Sesquiterpene	0.02
31.3	Cis-Thujanol	0.06
31.4	Linalool	4.63
32.2	Trans-p-Menth-2-en-1-ol	0.05
32.7	Bornyl formate + ε-Cadinene	0.05
33.1	Bornyl Acetate	0.08
34.0	Methyl Thymol Ether	0.03
34.2	β-Caryophyllene	2.64
34.5	Terpinene-4-ol	1.60
34.6	Carvacrol methyl ether	0.18
34.8	Aromadendrene	0.08
34.9	Hotrienol	0.04
35.1	Sesquiterpene	0.03
36.0	Sesquiterpene	0.01
36.1	Cis-p-Menth-2-en-1-ol	0.04
37.0	Allo-Aromadendren	0.05
38.4	α-Humulen	0.09
38.7	Sesquiterpene	0.02
39.0	Lavandulol	0.05
39.1	Verbenol	0.02
39.3	Neral	0.05
39.6	Sesquiterpene	0.04
40.0	γ-Muurolene	0.07
40.2	α-Terpineol	0.15
40.3	Borneol	1.75
40.6	Verbenone	0.17
40.8	Germacrene D	0.03
41.2	Carvenone	0.02
41.8	Sesquiterpene	0.02
42.0	α-Muurolene	0.05
42.2	β-Bisabolene	0.05
42.4	Bicyclogermacrene	0.07
43.0	Cis-Piperitol	0.02
43.4	Trans-Isopiperitenol	0.01
43.6	δ-Cadinene	0.09
43.7	γ-Cadinene	0.05
46.2	Nerol	0.02
47.4	Calamenene	0.01

48.3	Isopiperitenon	0.02
48.8	Geraniol	0.06
48.9	p-Cymene-8-ol	0.04
49.0	Thymyl Acetate	0.06
50.8	Myrcenol	0.03
51.1	Epi-Cubebol	0.01
51.5	Geranyl Butyrate	0.03
51.7	Neryl Methylbutyrate	0.02
55.5	Isocaryophyllene Oxide	0.01
56.0	Caryophyllene Oxide	0.16
56.6	1.10-Decanediol	0.08
57.4	Aromatic Compounds	0.01
57.8	Phenyl Butanone Isomer	0.07
59.1	Methyl Benzoate	0.02
62.0	Cuminol	0.02
62.5	Aliphatic Ester	0.02
63.3	Spathulenol	0.07
65.2	Eugenol	0.03
65.5	Isothymol	0.07
66.4	Thymol	46.84
67.1	Isocarvacrol	0.08
67.4	Sesquiterpenol	0.03
67.6	Carvacrol	4.58
68.3	Sesquiterpenol	0.01
68.5	α-Cadinol	0.02
69.0	Sesquiterpenol	0.03
	**TOTAL**	**99.99**

Table S4: *Thymus vulgaris* (CT thujanol) GCMS analysis

RT	Costituents	Percentage
5.0	2-Methyl Butanal	0.01
5.1	Isovaleraldehyde	0.03
5.2	Ethanol	0.07
6.3	2-Methylbutyrate	0.02
6.4	Tricyclene	0.02
6.5	Methyl Isovalerate	0.01
6.6	α-Pinene	1.67
6.7	α-Thujene	0.63
7.6	Camphene	0.22
7.9	Hexanal	0.01
8.8	β-Pinene	0.64
9.2	Sabinene	1.74
10.6	**β-Myrcene**	**4.41**
10.7	α-Phellandrene	0.06
11.3	**α-Terpinene**	**3.30**
11.7	2.3-Dehydro-1.8-Cineole	0.02
12.1	**Limonene**	**2.70**
12.2	Dodecan	0.03
12.5	1.8-Cineol	0.05
12.6	β-Phellandrene	0.64
13.0	2-Hexenal	0.01

13.7	Cis-β-Ocimene	0.01
14.3	**γ-Terpinene**	**5.93**
14.5	Trans-β-Ocimene	0.02
14.7	3-Octanon	0.04
15.0	Propanoate 4-Pentenyl	0.01
15.4	μ-Cymene	0.01
15.5	p-Cymene	1.06
16.1	Terpinolene	1.27
19.2	4-Pentenyl Butyrate	0.03
19.4	Hexyl Butyrate	0.02
19.9	1-Hexanol	0.02
21.5	1-Octen-3-yl Acetate	0.02
21.7	3-Hexen-1-ol	0.03
22.0	Hexyl Propanoate	0.01
22.4	3-Octanol	0.06
23.9	Compound Mw=150	0.04
25.0	α-p-Dimethylstyrene	0.01
25.3	Linalool cis-Oxide	0.02
25.5	Limonene cis-Oxide	0.01
25.8	1-Octen-3-ol	0.33
26.3	Limonene trans-Oxide	0.03
26.9	**Trans-Thujanol**	**22.71**
27.1	Linalol Trans-Oxide	0.03
28.4	Menthatrienic compound	0.02
29.0	Camphor	0.03
29.6	Compound Mw=152	0.09
30.0	β-Bourbonene	0.08
31.7	Cis-Thujanol	4.73
31.9	Linalool	9.45
32.4	Linalyl Acetate	1.59
32.8	Trans-p-Menth-2-en-1-ol	0.80
33.4	ε-Cadinene	0.03
33.7	Bornyl Acetate	0.05
34.5	β-Cubebene	0.05
34.7	β-Caryophyllene	1.83
35.2	Terpinene-4-ol	12.05
35.5	Cis-Dihydrocarvone	0.06
35.7	Sesquiterpene	0.04
36.2	Sesquiterpene	0.04
36.5	Trans-Dihydrocarvon	0.10
36.7	Cis-p-Menth-2-en-1-ol	0.37
37.4	Sesquiterpene	0.03
37.6	Sesquiterpene	0.04
39.0	α-Humulene	0.09
39.4	E-β-Farnesene	0.05
39.7	Cis-Piperitol	0.17
39.9	Neral	0.01
40.2	γ-Muurolene	0.05
40.7	Ocimenone	1.27
40.8	α-Terpineol	2.62
40.9	Borneol	0.21
41.4	Germacrene D	0.57
42.5	Neryl Acetate	0.23

42.8	Bicyclogermacrene	0.15
43.4	Pyranic Linalol Cis-Oxide	0.05
43.6	Trans-Piperitol	0.28
44.2	δ-Cadinene	0.07
44.3	Geranyl Acetate	0.07
44.8	Myrcenyl Acetate	3.96
46.2	Terpenic Ester	0.04
46.7	Nerol	0.02
47.0	Menthatriene Isomer	0.03
47.8	Myrcenyl Butyrate	0.06
48.0	Terpenic Acetate	0.08
48.2	Calamene + Menthatrienic compound	0.30
48.5	Terpenic Ester	0.72
48.7	Trans-Carveol	0.04
49.4	Geraniol	0.07
50.5	Ester Terpenique	0.04
51.4	Myrcenol	7.90
52.3	Aromatic Compound	0.20
53.1	Ester Myrcene	0.22
56.2	Isocaryophyllene Oxide	0.02
56.6	Caryophyllene Oxide	0.12
57.2	Aliphatic alcohol	0.01
59.7	Aliphatic Ester	0.03
60.2	Germacra-1.5-dien-4-ol	0.04
62.2	Cis-1.2-Dihydroxy-p-Menthe-2-en	0.04
62.9	10-Epi-γ-Eudesmol	0.03
63.9	Spathulenol	0.03
65.8	Eugenol	0.06
66.2	Eugenol	0.03
66.8	Thymol	0.29
68.2	Carvacrol	0.09
69.2	α-Cadinol	0.03
72.1	Caryophylla-3.7-Dien-6-ol	0.02
75.1	Sesquiterpenol	0.01
	**TOTAL**	**99.91**

Table S5: *Satureja montana* hydrolate GCMS analysis

RT	Costituents	Percentage
**18.6**	Verbenone	0.05
**21.8**	Thymol	13.88
**22.2**	Carvacrol	85.79
**41.7**	Palmidic acid. ethyl ester	0.06
**48.8**	Squalene	0,21

Table S6: *Citrus aurantium* var. *amara* hydrolate GCMS analysis

Costituents	Percentage
α-Pinene	0.03

Carvone	2.14
Cineole	0.03
Citronellol	2.14
p-Cymene	0.09
Geraniol	6.43
Limonene	0.21
Linalool	47.7
Myrcene	0.26
Nerol	1.93
Trans-β-Ocimene	0.12
α-Phellandrene	0.02
Terpinen-4-ol	0.21
α-Terpineol	13.83
Terpinolene	24.82
